# Impact of a Population Genomic Screening Program on Health Behaviors Related to Familial Hypercholesterolemia Risk Reduction

**DOI:** 10.1161/CIRCGEN.121.003549

**Published:** 2022-07-12

**Authors:** Laney K. Jones, Nan Chen, Dina A. Hassen, Megan N. Betts, Tracey Klinger, Dustin N. Hartzel, David L. Veenstra, Scott J. Spencer, Susan R. Snyder, Josh F. Peterson, Victoria Schlieder, Amy C. Sturm, Samuel S. Gidding, Marc S. Williams, Jing Hao

**Affiliations:** Geisinger, Danville, PA (L.K.J., N.C., D.A.H., M.N.B., T.K., D.N.H., V.S., A.C.S., S.S.G., M.S.W., J.H.).; University of Washington, Seattle (D.L.V., S.J.S.).; Georgia State University, Atlanta (S.R.S.).; Vanderbilt University Medical Center, Nashville, TN (J.F.P.).

**Keywords:** cholesterol, follow-up studies, hypercholesterolemia, lipoproteins, LDL, retrospective studies

## Abstract

Limited information is available regarding clinician and participant behaviors after disclosure of genomic risk variants for familial hypercholesterolemia (FH) from a population genomic screening program.

**Methods:**

We conducted a retrospective cohort study of MyCode participants with an FH risk variant beginning 2 years before disclosure until January 16, 2019. We analyzed lipid-lowering prescriptions (clinician behavior), medication adherence (participant behavior), and LDL (low-density lipoprotein) cholesterol levels (health outcome impact) pre- and post-disclosure. Data were collected from electronic health records and claims.

**Results:**

The cohort included 96 participants of mean age 57 (22–90) years with median follow-up of 14 (range, 3–39) months. Most (90%) had a hypercholesterolemia diagnosis but no specific FH diagnosis before disclosure; 29% had an FH diagnosis post-disclosure. After disclosure, clinicians made 36 prescription changes in 38% of participants, mostly in participants who did not achieve LDL cholesterol goals pre-disclosure (81%). However, clinicians wrote prescriptions for fewer participants post-disclosure (71/96, 74.0%) compared with pre-disclosure (81/96, 84.4%); side effects were documented for most discontinued prescriptions (23/25, 92%). Among the 16 participants with claims data, medication adherence improved (proportion of days covered pre-disclosure of 70% [SD, 24.7%] to post-disclosure of 79.1% [SD, 27.3%]; *P*=0.05). Among the 52 (54%) participants with LDL cholesterol values both before and after disclosure, average LDL cholesterol decreased from 147 to 132 mg/dL (*P*=0.003).

**Conclusions:**

Despite disclosure of an FH risk variant, nonprescribing and nonadherence to lipid-lowering therapy remained high. However, when clinicians intensified medication regimens and participants adhered to medications, lipid levels decreased.

Familial hypercholesterolemia (FH) is a common inherited disorder of cholesterol metabolism that leads to premature atherosclerotic cardiovascular disease. Approximately 17 500 deaths per year and 3% to 10% of heart attacks in people under 45 years of age are attributable to FH.^1^ Individuals with variants in FH-associated genes have triple the risk for atherosclerotic cardiovascular disease at any LDL (low-density lipoprotein) cholesterol (LDL-C) level^2^ due to lifelong exposure to cholesterol,^2^ Those with FH require early, aggressive, and sustained lipid-lowering therapy^3^ to reduce atherosclerotic cardiovascular disease rates.

Reports from many countries including the United States have shown underprescribing of lipid-lowering therapies for individuals with FH.^4–7^ However, some countries that have genomic screening programs for FH have signaled an improvement in both prescribing of and adherence to lipid-lowering therapies after disclosure of a result.^8^ In addition, pharmacogenomic information is available from these programs, which provides insights into lipid management. For example, a variant in the *SLCO1B1* gene is associated with muscle-related side effects of statin therapies and thus is a potential cause of nonadherence. However, the status of this variant in individuals with FH often remains unknown.^9–11^

Several health care systems in the United States and other countries have initiated population-based genomic screening programs.^12–15^ These programs provide FH diagnoses by identifying disease-causing variants in FH-associated genes. As genomic screening becomes more widespread to identify individuals with FH, it will be important to understand the impact of the return of these results on medication prescribing, adherence, and health outcomes such as lipid levels that are predictive of atherosclerotic cardiovascular disease risk.

We have previously reported on the initial 28 participants who received an FH result from the MyCode Community Health Initiative (MyCode), a population-based genomic screening program at Geisinger.^16^ This study suggested positive changes in clinician and participant behaviors after disclosure of a risk variant in an FH-associated gene to participants and their clinician.^17^ The objective of the current study is to use additional disclosed results from this program to evaluate clinician and participant behaviors after receiving a genomic risk result for FH and its impact on medication prescribing, medication adherence, and lipid level outcomes.

## Methods

Full description of the study methods is included in the Supplemental Material. The data that support the findings of this study are available from the corresponding author upon reasonable request. This study is part of the Rational Integration of Clinical Sequencing R01 project, which was approved by the Geisinger Institutional Review Committee; a waiver of the Health Insurance Portability and Accountability Act authorization was obtained.

## Results

### Demographics

A total of 96 participants had a variant in an FH-associated gene and met inclusion criteria from the 92 455 participant cohort. Mean age was 57 (SD, 17) years, 61 of 96 (63.5%) were female, and 33 of 96 (34.4%) had documented statin intolerance. Median length of follow-up from FH risk variant disclosure to the end of the study period was 14 months (range, 3–39). Before the genomic risk result disclosure, most participants, 86 of 96 (89.6%), had a diagnosis of hypercholesterolemia documented in the electronic health record, but no one (0%) had a specific diagnosis of FH (*International Classification of Diseases, Tenth Revision*, diagnostic code E78.01). After disclosure, 28 of 96 (29.2%) had the specific FH diagnosis code E78.01, added to their problem list by their clinician. Eighteen participants (18.8%) had a history of myocardial infarction or stroke. Seventy-seven participants (80.2%) had LDL-C values before disclosure with a mean (SD) of 152 (68) mg/dL. Among these, 10 of 77 (13.0%) achieved their LDL-C targets relevant to primary (LDL-C, <100 mg/dL) or secondary (LDL-C, <70 mg/dL) prevention. Table 1 details the demographics.

**Table 1.**
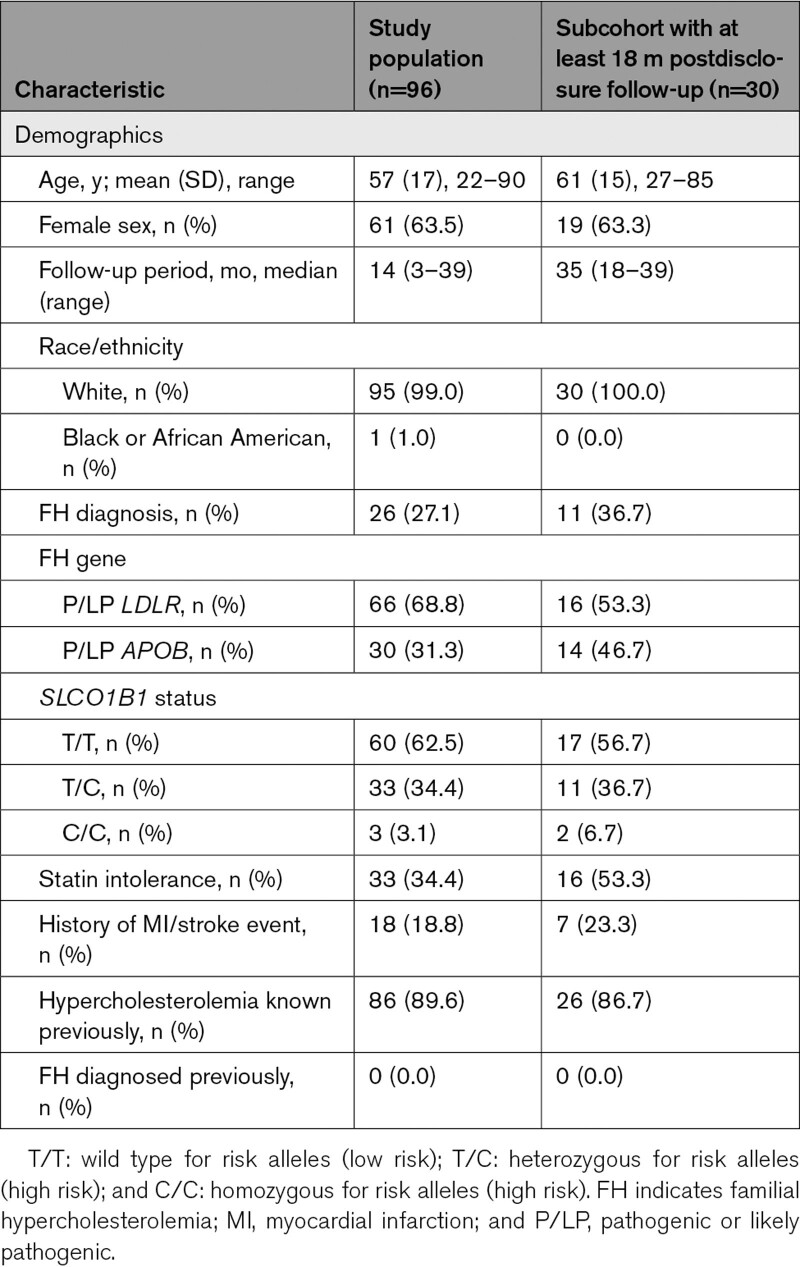
Participant Characteristics

### Clinician Behavior After Learning About an FH-Associated Genomic Risk Result for Their Participant

**Figure 1.**
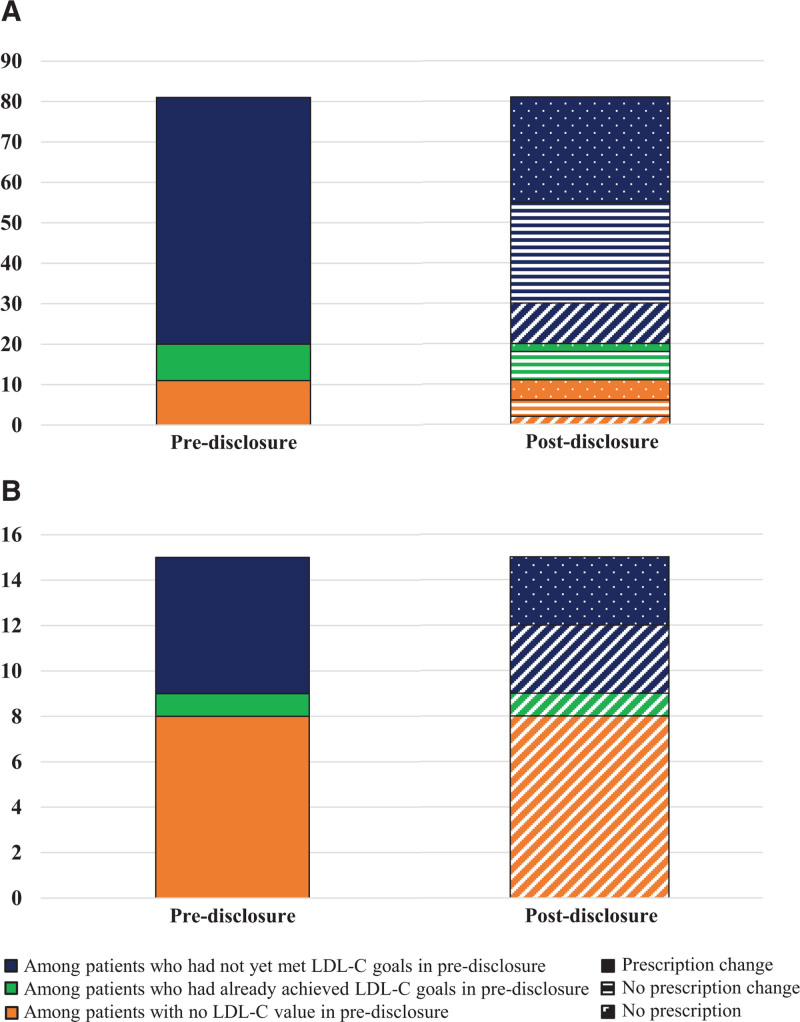
**Clinician behavior of prescription change. A**, Among patients with lipid-lowering therapy prescription pre-disclosure (n=81). **B**, Among patients without lipid-lowering therapy prescription pre-disclosure (n=15).

Clinicians wrote prescriptions for fewer participants after result disclosure (pre-disclosure, 81/96 [84.4%] versus post-disclosure, 71/96 [74.0%]; Table 2). Of these, 19 participants did not have an LDL-C value available for review in the electronic health record, 10 participants were at their LDL-C target and did not require medication intensification, and 67 participants were not yet at goal before disclosure. Clinicians made prescription changes post-disclosure in 36 of 96 (37.5%) participants, most of whom were among the 67 participants who had not yet achieved their LDL-C target (29/36, 80.6%). The most common change was intensifying the medication regimen (23/36, 63.8%) including use of a high-intensity statin or adding or switching to a prescription for ezetimibe or a PCSK9 inhibitor (n=6). All PCSK9 inhibitors prescribed were made after disclosure. Clinicians made no changes to participants’ medication regimens in 36 of 96 (37.5%). Clinicians discontinued lipid-lowering prescription for 12 of 96 participants, and 12 of 96 never had a prescription. Clinicians documented reasons for not prescribing or discontinuing medication in most (23/24, 95.8%) of these participants, including statin intolerance (n=8), pregnancy (n=6), fear of side effects (n=2), concerns regarding costs (n=3), no health insurance (n=1), active military (n=1), refusal to take a statin (n=1), and sought alternative treatment (n=1). About three-quarters of participants (70/96, 73%) had a lipid panel ordered by their clinician after learning about their FH result. Additional details on medications prescribed and laboratory orders are available in Table 2. Figure 1 demonstrates paths of clinician prescription changes from pre- to post-disclosure stratified by participants who started on therapy in predisclosure (n=81) and those who did not (n=15).

**Table 2.**
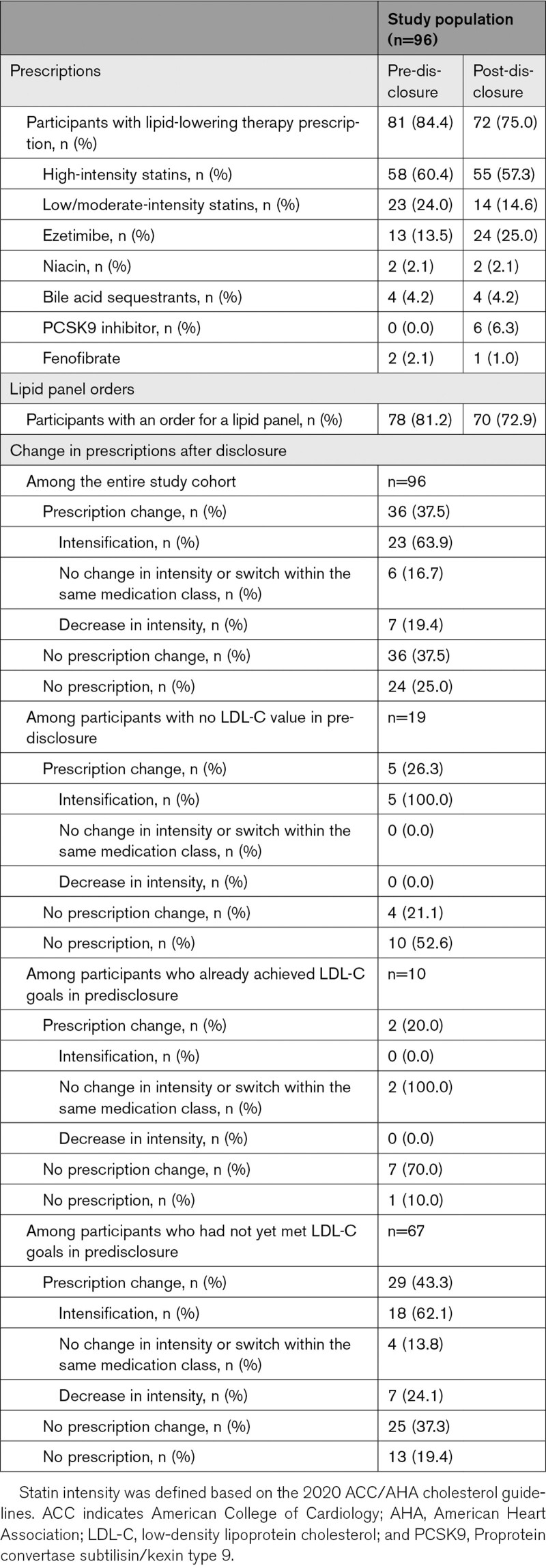
Clinician Behavior

In the multivariate regression analysis, only predisclosure LDL-C levels influenced clinician prescribing (Table S1). Clinicians were 14.6× more likely to change medication regimens or 22.9× more likely to issue no prescription in participants when LDL-C levels were >190 mg/dL compared with reference (LDL-C, <100 mg/dL; *P*=0.024 and *P*=0.023, respectively). No differences in prescribing behavior were observed in the 100 mg/dL <LDL-C 190 mg/dL or LDL-C <100 mg/dL groups.

### Participant Behavior After Learning About Their FH Genomic Risk Variant

Less than half (40/96, 41.7%) of the participants opted to complete a no-cost appointment with a genetic counselor to discuss their FH result, offered to all participants as part of the MyCode Genomic Screening and Counseling Program. About three-quarters (70/96, 72.9%) of participants had at least 1 lipid panel drawn after learning their result, which was approximately equivalent to the proportion that had a panel drawn before learning the result (77/96, 80.2%). In the subset of 16 participants with continuous Geisinger Health Plan coverage, only 4 of 16 (25.0%) had changes post-disclosure in the medication regimens. There was no change in the participants who were adherent to their medication regimens (obtaining a proportion of days covered of ≥80%), but mean medication adherence rates improved (proportion of days covered, mean [SD]: pre-disclosure, 70.0% [24.7%] versus post-disclosure, 79.1% [27.3%]; *P*=0.05). Additional details on medication adherence are available in Table 3. Statin intolerance was present in a substantial proportion of participants (33/96, 34.4%), but *SLCO1B1* variant status showed no correlation with the documentation of intolerance (data not shown).

**Table 3.**
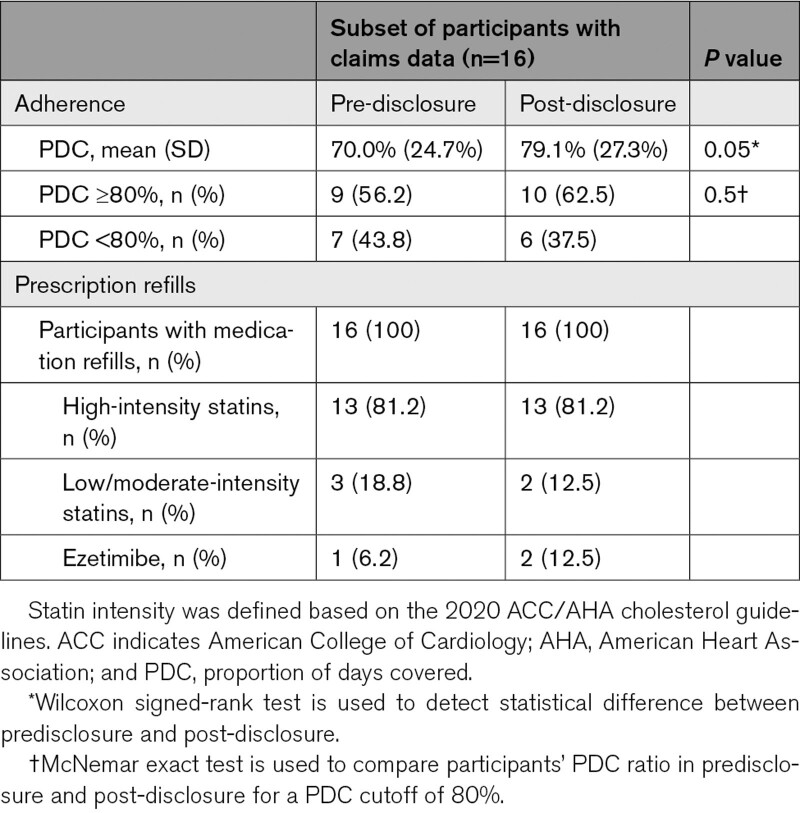
Participant Behavior

### Impact of Clinician and Participant Behaviors on Lipid Levels

**Figure 2.**
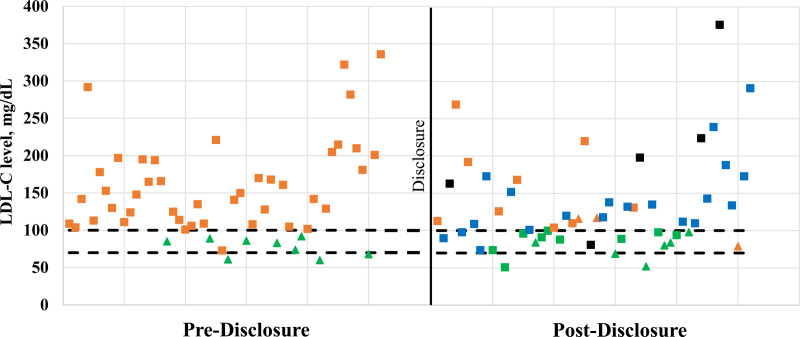
**LDL (low-density lipoprotein) cholesterol (LDL-C) levels pre- and post-disclosure.** *Decreased and increased change in post-disclosure is only counted as valid change when the percentage of LDL-C level change is ≥8%. †LDL-C value is <100 mg/dL for primary prevention or <70 mg/dL for secondary prevention.

In a subset of 52 participants with predisclosure and postdisclosure lipid values (Table 4), LDL-C was significantly reduced after disclosure (LDL-C, mean [SD]: pre-disclosure, 147 [64] mg/dL versus post-disclosure, 132 [63] mg/dL; *P*=0.003; a 7.7% reduction in mean level). One-third (15/52, 28.8%) of participants achieved LDL-C goal after disclosure. Figure 2 with each scatter plot representing one participant demonstrates how participants move with relative to their LDL-C goal from pre- to post-disclosure, as well as the magnitude of lipid level change. Having a high (LDL-C, ≥190 mg/dL) predisclosure LDL-C level (*P*=0.034) or having a history of myocardial infarction/stroke (*P*=0.043) was associated with participants being less likely to achieve LDL-C target levels (Table 5). Clinician prescribing behavior was not associated with participants achieving LDL-C target goals (Table 5). About a third of participants (6/20, 30%) who had prescription changes made by their clinician were able to achieve LDL-C goals (Table 5). While some participants achieve LDL-C goals with no changes, most did not (Table 5).

**Table 4.**
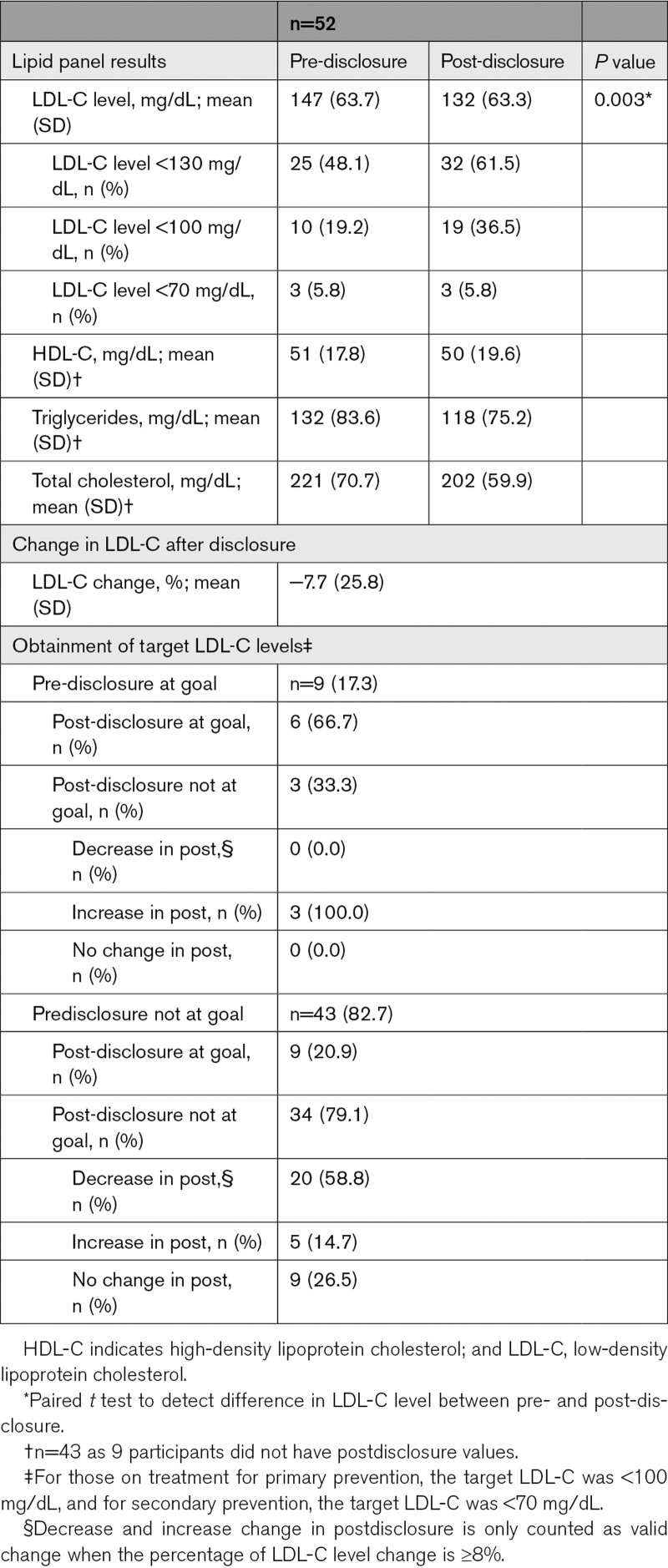
Lipid Levels

**Table 5.**
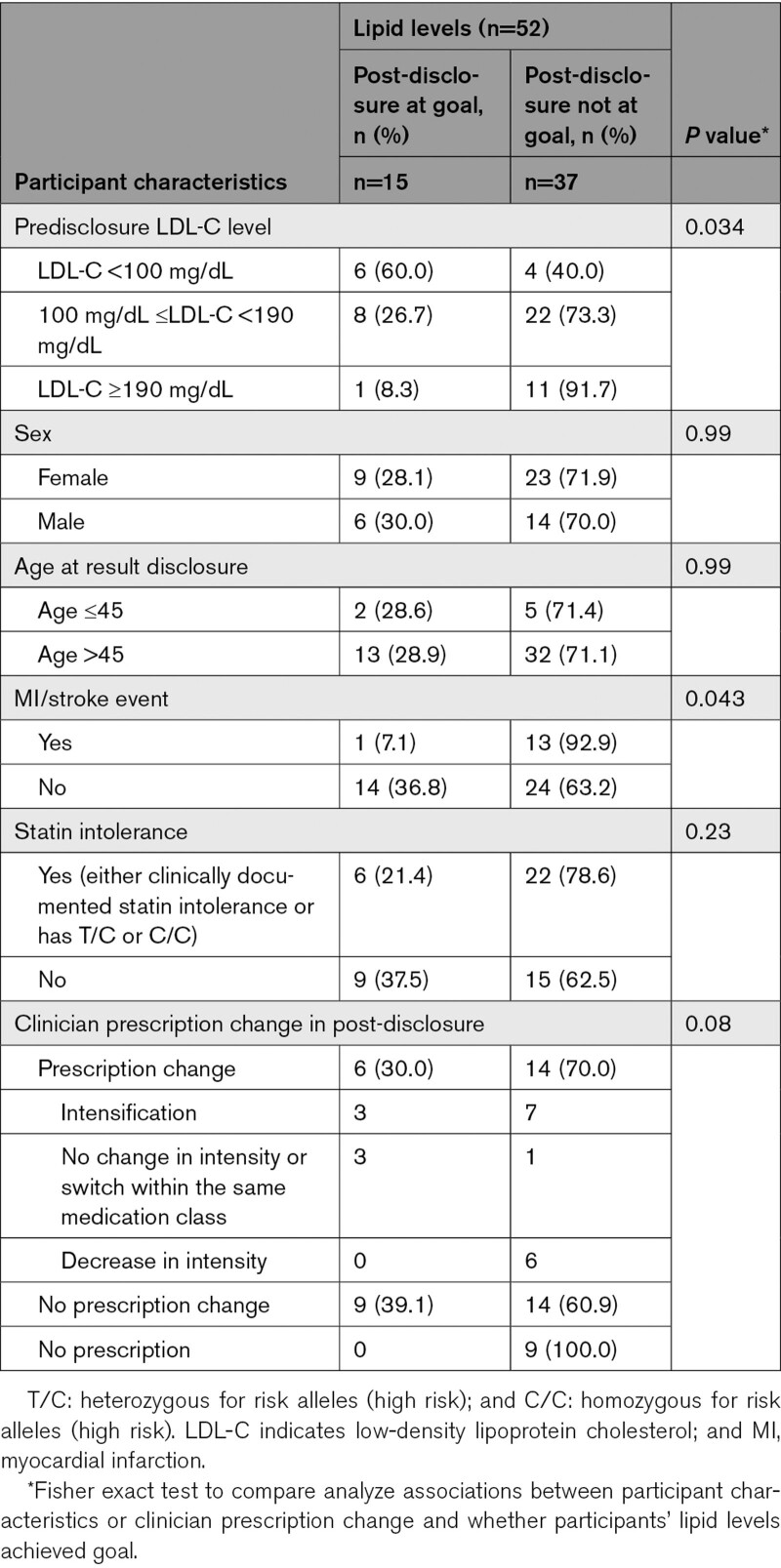
Participant Characteristics, Clinician Prescribing Behavior Change and Participant Lipid Levels

### Sensitivity Analysis

Consistent with observation in the entire cohort, among the subcohort of patients with at least 18 months of postdisclosure follow-up, clinicians wrote prescriptions for fewer participants in the post-disclosure period but did make prescription changes for about 40% of the participants mainly among those have not yet met LDL-C goal in predisclosure. However, in the subcohort, the majority of the prescription changes were intensification (10/12, 83%) versus in the entire cohort (23/36, 64%; Table S2). The lipid level also showed a reduction trend in the subcohort post-disclosure; however, the change was not significant (LDL-C, mean [SD]: pre-disclosure, 155 [73] mg/dL versus post-disclosure, 142 [78] mg/dL; *P*=0.23). The subcohort had fewer participants at goal at the end of follow-up compared with the entire cohort (Table S3). These differences in outcomes might have to do with a harder-to-treat population of the subcohort with a higher percentage of participants with pathogenic or likely pathogenic *APOB* (14/30, 47% versus 30/96, 31%) and history of myocardial infarction/stroke (7/30, 23% versus 18/96, 19%) and a much higher statin intolerance rate (16/30, 53% versus 33/96, 34%; Table 1).

## Discussion

We studied the impact of disclosing genomic risk variants in FH-associated genes to unselected individuals who participated in a population genomic screening initiative. We found substantial changes to both clinician and patient behavior after return of FH genomic risk result. After disclosure, clinicians intensified medication regimens in about a third of the participants who did not achieve LDL-C targets before disclosure and in a quarter of all participants. Clinicians had documented various reasons for decisions regarding switches in the statin medication class and discontinuation of medications. Approximately 42% of participants sought follow-up to discuss their genomic risk variants in an FH-associated gene with a genetic counselor. Only 62.5% of participants had good adherence to their lipid-lowering therapies at the end of the observation period, but this result showed a positive trend compared with predisclosure. There was no relationship of documented statin intolerance to variants in *SLCO1B1.* About one-third of the participants were able to achieve the LDL-C goal and had an average 7.7% reduction in LDL-C post-disclosure. Having a high LDL-C level >190 mg/dL before disclosure or a history of myocardial infarction/stroke are risk factors that made participants less likely to achieve their lipid level goal. We performed a sensitivity analysis of those participants with >18 months of follow-up and observed consistent trends in this subcohort compared with the entire cohort in terms of prescription change and lipid outcomes. However, this cohort tended to have higher levels of statin intolerance and higher percentage of cardiovascular events; more prescription intensifications were made, but worse lipid outcomes were achieved.

While most individuals had a diagnosis of hypercholesterolemia, no participant had a specific diagnosis of FH on their problem list before return of the genomic risk result. This result is not unexpected, as data from a recent meta-analysis show that the prevalence of FH is unknown in 91% of countries worldwide^18^ and only 10% of individuals are aware of their diagnosis. There are a variety of reasons for the underdiagnosis of FH^19^; current work in the field is focused on using automated methods to identify individuals with FH by combining various data sets including clinical, claims, and genetic data.^20–22^ However, after the return of the genomic risk result for FH, clinicians documented the FH diagnosis (*International Classification of Diseases, Tenth Revision*, code E78.01) in 29% of their participants, which, while an improvement, still means over two-thirds do not have the diagnosis documented in the electronic health record. Further exploration of clinician reluctance to modify the problem list, a key resource for care coordination, is warranted. A potential intervention to consider is the implementation of a lifetime genetic record that populates the problem list with key genetic diagnoses that require longitudinal management.

Our results suggest that the confirmation of diagnosis of FH is a barrier to FH care; however, its importance in comparison to other major barriers such as managing statin-associated muscle symptoms (real or perceived), clinician and patient knowledge gaps related to FH care, access to medications such as PCSK9 inhibitors, among others is unknown. The National Lipid Association has published guidance on how to effectively manage statin-associated muscle symptoms, while still reducing cardiovascular disease burden.^23^ Clinician and patient knowledge related to FH impacts care from identification to treatment.^24–27^ Once identified, access to treatment can be difficult. Low prescribing of PCSK9 inhibitors due to access problems is consistent with a large cohort study conducted by the Family Heart Foundation (previously the FH Foundation).^28^

An interesting and concerning observation in our study was the decrease in the number of participants receiving prescriptions for lipid-lowering therapies after clinicians and participants learned about the FH genomic risk result. This is similar to what was reported in our pilot study,^17^ although others have seen an increase in the number of prescriptions after return of a genomic risk result.^13^ However, these changes may be a reflection of trends in prescription behavior over time independent of FH genomic risk disclosure, and the short follow-up time (minimum 3 months) limited our ability to detect longer term changes. One potential driver of this phenomenon is that clinicians were revisiting participants’ prescriptions after learning their genomic risk for FH and uncovered side effects or other issues their participants are having related to initiation and adherence to lipid-lowering therapies, in particular to statin therapy. In fact, unlike previous work, we were able to describe reasons for medication discontinuation or nonprescribing where we reveal participant-reported side effects from medication was the primary reason, although some medically appropriate discontinuations were also noted (eg, pregnancy).

Similar to our work, Hollands et al^29^ found, through semistructured interviews with participants being tested for FH, that the impact of learning about a genetic FH diagnosis had a relatively small impact on changing participants’ health behaviors. In our findings, ≈42% of participants sought follow-up with a genetic counselor, which could be due to short follow-up time or due to learning about their result from their primary care clinician. In comparison, Geisinger analyzed genetic counseling visits across return of genomic risk results for all conditions returned through MyCode and found a higher uptake of genetic counseling visits (55.3%) in other conditions.^30^ However, when pre- and postdisclosure genetic counseling was integrated into a multidisciplinary lipid clinic for FH at Geisinger, almost all participants opted for the visit.^25^ One potential reason for lower uptake of genetic counseling in this study could be the requirement of scheduling and completing an additional visit, supporting the integration of genetic counseling services into other specialty care. Use of chronic medications, such as lipid-lowering therapies, can be difficult for participants to initiate and sustain. Individuals with FH have reported low (63%) medication adherence rates when surveyed.^31^ We found similar rates of adherence to lipid-lowering therapies as others.^32^ We did not find any relation of side effects with pharmacogenomic information, and recent research suggests statin side effect complaints may be related to participant perception as opposed to statin side effect complaints associated with physiological or pharmacogenetic factors as currently understood.^33^

Another important finding from our work is 30% of participants with a prescription change were able to achieve LDL-C goals, even with a relatively short follow-up time. Others have found similar achievement of LDL-C targets (21%) after return of a genomic risk result.^34^ The Dutch FH Program, which aimed to identify all individuals in the Netherlands with FH through assessment of both clinical and genetic markers and conducted cascade screening on at-risk individuals, found a 10.3% reduction in LDL-C in those on treatment,^8^ which is higher than the 7.7% reduction in our findings. However, the Dutch program focused on a different cohort, first identifying probands through lipid clinics and then cascade testing at-risk relatives, and also observed a significant increase in prescribing treatment to individuals with FH.^13^ We found that participants with LDL-C (>190 mg/dL) and those with previous cardiovascular events had more difficulty achieving LDL-C targets, which is consistent with literature.^35,36^ This observation could mean that these individuals had higher baseline LDL-C levels with lower LDL-C thresholds, more severe disease, were intolerant to statin medications, or may require more intensive therapies such as PCSK9 inhibitors to achieve adequate reduction in cardiovascular risk. The findings of this study have relevance to the initiation phase of implementation. It indicates that clinician and participant health behavior related to FH care should be a subject of future studies to understand how to address and overcome these barriers to care.

In addition to clinician- and patient-level interventions, there is a need for system-level and policy interventions to improve FH care. Barriers identified in this study might be part of a larger systemic problem related to lipid management including the removal of Healthcare Effectiveness Data and Information Set metrics for LDL-C in 2013. Further research exploring multilevel interventions to improve lipid management are needed. These could include implementation of guidelines for lipid management that align with and support clinician workflow, use of quality improvement programs within health care systems, patient-focused interventions to improve awareness of FH, and policy interventions that address system barriers to lipid management. These interventions must be coupled with robust measurement tools to assess their effectiveness and should include both patient- and provider-centric measures that include outcomes related to service and satisfaction.

### Limitations

There are a few limitations to this work. First, there was no comparison group to discern other factors that might have impacted care other than the introduction of the genomic risk result for FH. The impact of time trends effect was not evaluated, that is, some of the changes might occur as trends over time independent of FH genomic risk disclosure. A separate study at Geisinger to investigate longitudinal trends in lipid levels over the 10-year time span is underway. Second, even though some individuals had longer follow-up, most only had ≈1 year of follow-up. This may have impacted our results as general hypercholesterolemia is typically cared for on a yearly basis with annual lipid measurement, which may have explained the absence of lipid values in 20% of the population. Also, initiatives to improve the medication reconciliation process, which is a known problem at Geisinger and other US health care systems, may have accounted for the larger apparent discontinuation of prescriptions after result disclosure but could reflect more accurate determination of actual medication use, that is, identifying participants not taking medication who were misclassified as on medication based on errors in reconciliation.

### Conclusions

Changes in clinician behavior, patient behavior, and intermediate outcomes were seen after the disclosure of genomic information on FH. Clinicians intensified lipid-lowering regimen. Patients sought genetic counseling, had lipid panels drawn, and were more adherent to their lipid-lowering medications. As a result, there was a reduction in lipid levels and increased attainment of target cholesterol goals, though a minority achieved the LDL-C goal, meaning a significant care gap remains. Nonprescription by providers and nonadherence to lipid-lowering therapy by patients remained high. Future studies on clinician behavior should focus on prescribing; studies on patients should focus on nonadherence to lipid-lowering therapies, with a goal of improving the percentage of patients getting to LDL-C treatment goal.

## Article Information

### Sources of Funding

This study is funded by the National Institutes of Health/National Human Genome Research Institute (grant number R01 HG009694-01; project title: Rational Integration of Clinical Sequencing).

### Disclosures

L.K. Jones is a consultant for Novartis, and cochair of the Health Quality and Research Committee of the National Lipid Association. Dr Peterson serves on the advisory board for Natera. A.C. Sturm is funded by National Institutes of Health, NHLBI R01, 5R01HL148246-02 (IMPACT-FH [Identification Methods Patient Activation and Cascade Testing for Familial Hypercholesterolemia]), the Chair of the Advisory Board for the All of Us Genetic Counseling Resource, SAB Member of The FH Foundation, and consultant for 23andMe, Invitae. Dr Gidding is a consultant for Esperion. D.N. Hartzel is a part-time contractor for Tempus. The other authors report no conflicts.

### Supplemental Material

Supplemental Methods

Tables S1–S4

References ^3,30,37–39,40–43^

## Supplementary Material


