# Supplementary material for: Impact of a Population Genomic Screening Program on Health Behaviors Related to Familial Hypercholesterolemia Risk Reduction
Source: Circ Genom Precis Med. 2022 Jul 12;15(5):e003549. doi: 10.1161/CIRCGEN.121.003549 (PMC9584046; doi:10.1161/CIRCGEN.121.003549)
Supplement: Supplementary file 1 [file hcg-15-e003549-s001.pdf]

SUPPLEMENTAL MATERIAL

SUPPLEMENTAL METHODS

Study Setting

This study was conducted at Geisinger, an integrated health care system serving central and northeastern Pennsylvania. Geisinger has hospital and ambulatory clinics, known as Geisinger Clinic, and a health insurance plan, Geisinger Health Plan (GHP). Approximately 30% of Geisinger Clinic participants are also GHP members. In MyCode, participant-participants provide samples which undergo exome sequencing. The MyCode research protocol has been detailed in previous publications.[37-39] Medically actionable variants associated with Mendelian disease are disclosed to both primary care clinicians (clinicians) and participants to inform future care. Results disclosure began in May 2015. Pharmacogenomic information (i.e., *SLCO1B1* variant status) is not currently returned to primary care clinicians and participants in MyCode.

Study cohort

The study population includes all adult MyCode participants from the 92,455 cohort, with variants in FH-associated genes, disclosed by the Genomic Screening and Counseling Program.[37] The study population had a significantly higher proportion of European ancestry and non-Hispanic ethnicity, older median age, and higher comorbidity index when compared to active Geisinger participants.[30] Multiple sources of health data, described in full below, were reviewed for a period beginning 2 years prior to the participant receiving a result and ending January 16, 2019. Individuals were excluded from data analysis if they: 1) withdrew from MyCode, 2) died, or 3) only had self-reported medication data available in the electronic health record (EHR).

Study Design

This is a retrospective observational study to compare clinician and participant behaviors and health outcomes before and after disclosure of a genomic risk variant in an FH-associated gene. The main outcomes are 1) clinician behavior, 2) participant behavior, and 3) impact of behavior on lipid levels, as defined below.

Data sources

The main data sources were the Geisinger EHR and GHP prescription claims data. Discrete EHR data were extracted through automated methods and included demographics, problem list diagnoses including hypercholesterolemia and FH (ICD-10 diagnostic code E78.01), lipid-lowering therapy prescriptions, lipid panels, and documentation of statin intolerance which is defined as statin allergy or documentation in a clinic note. Manual chart review was performed to validate and supplement non-discrete data including reasons for discontinuing lipid-lowering therapies. Prescription fill data from the EHR and claims data were used to supplement the EHR prescription information prescribed by non-Geisinger clinicians. Impact of *SLCO1B1* variant status obtained from MyCode exome sequencing data was evaluated.

To address possible missing health utilization data in the EHR, we evaluated and assessed the completeness of prescriptions and lipid panel laboratory data obtained from the EHR. We compared prescription and lipid panel utilization information in claims data, in a subset of the population who are GHP members and have at least 1 year of coverage, with what we obtained using the EHR supplemented with prescription adjudication data from community pharmacy among the same subset population. The analysis of the GHP subset showed that no missingness in prescription data was observed in the EHR supplemented with prescription fill data from community pharmacies; the discrepancy in lipid panel utilization was minimal (<5%). Given the high concordance, only EHR supplemented with prescription fill data were used for the remainder of the analyses.

Study Outcomes

Clinician behavior after learning about a FH-associated genomic risk variant for their participant

Clinician behavior was assessed pre- and post-disclosure by measuring the change in prescriptions for lipid-lowering therapies and in laboratory orders for a lipid panel. Medication prescriptions were categorized as follows: 1) prescription change—which is sub-categorized into intensification, no change in intensity or switch within the same medication class (only applicable to statin medications), and decrease in intensity, 2) no prescription change, and 3) no prescription. Documented reasons for discontinuation or no prescription were captured. Intensification was defined as the addition of medication therapy or increase in dose of current medications. No change in intensity or switch within the same medication class was defined as an increase or decrease in the dose of a statin or change in statin prescribed but not change in intensity based on the 2018 cholesterol guideline list of statin intensity. Decrease in intensity was defined as a removal of medication therapy or decrease in dose of current medications. No prescription change was defined as no change in medications prescribed from pre to post disclosure. No prescription was defined as no lipid lowering medications data was available in the Geisinger EHR.

We examined several participant covariates' association with the clinicians' behavior of prescription change post-disclosure: sex, age (stratified as age≤45 and age >45), pre-disclosure LDL-C level (stratified as 3 groups: LDL-C < 100 mg/dL, 100 mg/dL ≤ LDL-C < 190 mg/dL, and LDL-C ≥ 190 mg/dL), prior myocardial infarction (MI) and stroke, and statin intolerance. The pre-disclosure LDL-C value was defined as the most recent LDL-C drawn prior to the disclosure of the FH-associated variant.

Participant behavior after learning about their genomic risk variant for FH

Participant behavior was measured by participants' utilization of healthcare services relevant to FH management including completed visit with a genetic counselor, having a lipid panel result, and medication adherence. Among the subset of participants with claims data available (i.e. had continuous GHP prescription coverage (with no more than a 60-day gap of membership coverage during the observation period) and at least one prescription refill in pre- and post-disclosure) participant behavior was measured by medication refills and adherence in the pre- and post-disclosure periods. Medication adherence was measured by proportion of days covered (PDC), which is calculated as the ratio between the number of days covered by the medication and the length of the observation period.[40] A PDC of 80% and above was considered adherent to lipid-lowering therapies.[41]

Impact of clinician and participant behavior on lipid levels

The impact of clinician and participant behaviors after disclosure of an FH risk variant on lipid levels was evaluated in participants who had both pre- and post-disclosure LDL-C values available for review. Post-disclosure LDL-C was defined as the last LDL-C drawn before the end of the study period. To reflect the impact of clinician and participant behavior on LDL-C levels, in this analysis, we further restricted post-disclosure LDL-C to include those that reflect the most recent prescription, i.e. LDL-C level had to be tested at least 4 weeks after changing the prescription. We categorized change from pre- to post- disclosure in LDL-C value into 2 categories: 1) at goal and 2) not at goal. LDL-C goal was either an LDL-C value <100 mg/dL for primary prevention or <70 mg/dL for secondary prevention.[3] The decrease and increase of LDL-C level was considered a change when LDL-C level changed at least 8% between values.[42, 43]

We examined the association between covariates including clinician prescribing behavior change, the participants' lipid levels, and participant characteristics including sex, age, pre-disclosure LDL-C (stratified as the 3 groups noted above), prior event of MI and stroke, and

statin intolerance (where we incorporated both clinically documented statin intolerance and pharmacogenomics, i.e. *SLCO1B1* variant status).

Sensitivity Analysis in Sub-cohort

For considerations of the potential influence on the validity of outcomes in case of short post-disclosure follow up period, we conducted a sensitivity analysis to measure the key outcomes in a subset of the study cohort who had “sufficient” post-disclosure period which is defined as at least 18 months. We measured the clinician behavior focusing on change of prescriptions for lipid-lowering therapies and the impact of behavior on lipid levels. We did not analyze the participant adherence in this sub-cohort due to too small a sample size.

Statistical Analysis

Descriptive analyses were conducted to describe demographics of the study cohort and outcome measures. Outcome measures of continuous variables between pre- and post-disclosure were compared using Wilcoxon signed rank test, paired t-test or Wilcoxon exact signed rank test as appropriate. Categorical variables were compared between pre- and post-disclosure using McNemar exact test. Multinomial logistic regression was used to examine the association between patients’ characteristics and clinician behavior of prescription change in post-disclosure. Fisher exact test was used to analyze associations between co-variates including clinician prescribing behavior and whether participants’ lipid levels achieved goal.

Supplemental Table I. Multinomial Logistic Regression Models on Factors Associated with Clinician Prescription Behavior

**Study cohort—participants who have pre-disclosure LDL-C value (N = 77)**						
**Relative risk ratio**						
	100<= pre_LDL-C < 190	pre_LDL-C >= 190	Age >45	Male	Had MI/Stroke	Had statin intolerance
Prescription change	4.894	**14.556**	0.933	2.363	3.261	2.492
No Prescription	1.952	**22.874**	0.764	0.568	2.326	0.603
**p-value:**						
	100<= pre_LDL-C < 190	pre_LDL-C >= 190	Age >45	Male	Had event	Had statin intolerance
Prescription change	0.086	**0.024**	0.929	0.186	0.122	0.133
No Prescription	0.578	**0.023**	0.769	0.570	0.433	0.564

No Prescription Change is used as the comparison group, the results show statistics comparing Prescription Change and No Prescription Change, and comparing No Prescription with No Prescription Change group.

Supplemental Table II. Clinician behavior (sub-cohort of participants with at least 18m post-disclosure follow-up)

	Sub-cohort (n = 30)	
**Prescriptions**	Pre-disclosure	Post-disclosure
Participants with lipid-lowering therapy prescription, n (%)	25 (83.3)	21 (70.0)
High intensity statins, n (%)	18 (60.0)	16 (53.3)
Low/moderate intensity statins, n (%)	7 (23.3)	3 (10.0)
Ezetimibe, n (%)	5 (16.7)	10 (33.3)
Niacin, n (%)	1 (3.3)	1 (3.3)
Bile acid sequestrants, n (%)	3 (10.0)	2 (6.7)
PCSK9 inhibitor, n (%)	0 (0.0)	3 (10.0)
Fenofibrate	1 (3.3)	1 (3.3)
**Change in prescriptions after disclosure**		
Among the entire sub-cohort		N=30
Prescription change, n (%)		12 (40.0)
Intensification, n (%)		10 (83.3)
No change in intensity or switch within the same medication class, n (%)		0 (0.0)
Decrease in intensity, n (%)		2 (16.7)
No prescription change, n (%)		9 (30.0)
No prescription, n (%)		9 (30.0)
Among participants with no LDL-C value in pre-disclosure		N=4
Prescription change, n (%)		1 (25.0)
Intensification, n (%)		1 (100.0)

No change in intensity or switch within the same medication class, n (%)	0 (0.0)
Decrease in intensity, n (%)	0 (0.0)
No prescription change, n (%)	1 (25.0)
No prescription, n (%)	2 (50.0)
Among participants already achieved LDL-C goals in pre-disclosure	N=4
Prescription change, n (%)	0 (0.0)
Intensification, n (%)	0 (0.0)
No change in intensity or switch within the same medication class, n (%)	0 (0.0)
Decrease in intensity, n (%)	0 (0.0)
No prescription change, n (%)	4 (100.0)
No prescription, n (%)	0 (0.0)
Among participants had not yet met LDL-C goals in pre-disclosure	N=22
Prescription change, n (%)	11 (50.0)
Intensification, n (%)	9 (81.8)
No change in intensity or switch within the same medication class, n (%)	0 (0.0)
Decrease in intensity, n (%)	2 (18.2)
No prescription change, n (%)	4 (18.2)
No prescription, n (%)	7 (31.8)

Statin intensity was defined based on the 2020 ACC/AHA Cholesterol Guidelines.

Supplemental Table III. Lipid levels (sub-cohort of participants with at least 18m post-disclosure follow-up)

	n=20		
**Lipid Panel Results**	Pre-disclosure	Post-disclosure	P value
LDL-C level, mg/dL, mean (SD)	155 (73.1)	142 (78.4)	0.23*
LDL-C level < 130 mg/dL, n (%)	8 (40.0)	12 (60.0)	
LDL-C level < 100 mg/dL, n (%)	5 (25.0)	6 (30.0)	
LDL-C level < 70 mg/dL, n (%)	2 (10.0)	1 (5.0)	
HDL-C, mg/dL, mean (SD) †	58 (22.3)	56 (25.4)	
Triglycerides, mg/dL, mean (SD) †	125 (92.7)	107 (64.2)	
Total cholesterol, mg/dL, mean (SD) †	235 (86.6)	209 (65.1)	
**Change in LDL-C after disclosure**			
LDL-C change, %, mean (SD)	-2.5 (36.7)		
**Obtainment of target LDL-C levels ‡**			
**Pre-disclosure at goal, n (%)**	N=4 (20.0)		
Post-disclosure at goal, n (%)	2 (10.0)		
Post-disclosure not at goal, n (%)	2 (10.0)		
decrease in Post §, n (%)	0 (0.0)		
increase in Post, n (%)	2 (100.0)		
no change in Post, n (%)	0 (0.0)		
**Pre-disclosure not at goal, n (%)**	N=16		
Post-disclosure at goal, n (%)	1 (6.3)		
Post-disclosure not at goal, n (%)	15 (93.8)		
decrease in Post §, n (%)	10 (66.7)		
increase in Post, n (%)	3 (20.0)		
no change in Post, n (%)	2 (13.3)		

* Paired t test to detect difference in LDL-C level between pre- and post-disclosure.
† Two participants did not have post-disclosure values (N=18).
‡ For those treatment for primary prevention the target LDL-C was < 100 mg/dL and for secondary prevention the target LDL-C was < 70 mg/dL

§ Decrease and increase change in post-ROR is only counted as valid change when % of LDL-C level change is >=8%.

Supplemental Table IV. Individual level data on pre- and post-disclosure prescription and LDL-C change

Case	Follow-up period at least 18 months (Y/N)	Statin intolerance (Y/N)	History of MI or Stroke event (Y/N)	Pre-disclosure Prescription 1	Pre-disclosure Prescription 2	Pre-disclosure Prescription 3	Post-disclosure Prescription 1	Post-disclosure Prescription 2	Post-disclosure Prescription 3	Change in prescriptions after disclosure	LDL-C Goal status pre-disclosure*	LDL-C Goal status post-disclosure*
1	Y	N	Y	ROSUVASTATIN 40 MG	NIACIN ER 1000 MG		ROSUVASTATIN 40 MG	NIACIN ER 1000 MG	EZETIMIBE 10 MG	Prescription change: intensification	Pre not on goal	Post not on goal, decrease in Post
2	Y	Y	N	ATORVASTATIN 40 MG	COLESEVELAM HCL 625 MG		EVOLOCUMAB 420MG			Prescription change: intensification	NA	NA
3	N	N	N	ROSUVASTATIN 40 MG	NIACIN 1000MG		ROSUVASTATIN 40 MG	NIACIN 1000MG	EZETIMIBE 10MG	Prescription change: intensification	NA	NA
4	N	N	N	ATORVASTATIN 20 MG			ATORVASTATIN 20 MG			No prescription change	Pre not on goal	Post on goal
5	N	Y	N	ATORVASTATIN 80MG			ROSUVASTATIN 40 MG	EZETIMIBE 10MG		Prescription change: intensification	NA	NA
6	N	N	Y	ROSUVASTATIN 40MG	EZETIMIBE 10MG		ROSUVASTATIN 40MG	EZETIMIBE 10MG		No prescription change	Pre not on goal	Post not on goal, decrease in Post
7	N	Y	N	ATORVASTATIN 80 MG			ATORVASTATIN 40 MG			Prescription change: No change in intensity	Pre on goal	Post on goal
8	Y	Y	Y	ATORVASTATIN 80MG			ATORVASTATIN 80MG	EZETIMIBE 10MG		Prescription change: intensification	Pre not on goal	Post not on goal, decrease in Post
9	N	Y	N	ROSUVASTATIN 40MG			SIMVASTATIN 40MG			Prescription change: decrease in intensity	Pre not on goal	Post not on goal, no change in Post
10	N	N	Y	ATORVASTATIN 80MG	EZETIMIBE 10MG		ATORVASTATIN 80MG			Prescription change: decrease in intensity	Pre not on goal	Post not on goal, decrease in Post
11	Y	Y	N	ATORVASTATIN 10MG			ATORVASTATIN 10MG	EZETIMIBE 10MG		Prescription change: intensification	Pre not on goal	Post not on goal, decrease in Post
12	N	N	N	ATORVASTATIN 20MG			ATORVASTATIN 20MG			No prescription change	NA	NA

13	Y	Y	N	ROSUVASTATIN 40MG	FENOFIBRATE 54MG	EZETIMIBE 10MG	ROSUVASTATI N 40MG	FENOFIBRAT E 54MG	EZETIMIBE 10MG	No prescription change	Pre not on goal	Post not on goal, no change in Post
14	N	Y	N	ATORVASTATIN 10MG			ATORVASTATI N 20MG			Prescription change: No change in intensity	Pre not on goal	Post on goal
15	Y	N	N	ATORVASTATIN 40MG			ATORVASTATI N 40MG			No prescription change	Pre on goal	Post not on goal
16	Y	Y	N	ROSUVASTATIN 10MG	EZETIMIBE 10MG					No prescription	NA	NA
17	N	Y	Y	PRAVASTATIN 40MG			PRAVASTATIN 40MG			No prescription change	Pre not on goal	Post not on goal, no change in Post
18	Y	N	Y	ATORVASTATIN 80MG			ATORVASTATI N 80MG			No prescription change	Pre not on goal	Post not on goal, increase in Post
19	Y	Y	N	SIMVASTATIN 20MG			SIMVASTATIN 20MG			No prescription change	Pre on goal	Post not on goal
20	Y	Y	Y	ATORVASTATIN 80MG			ATORVASTATI N 80MG	EZETIMIBE 10MG	COLESEVELA M 625MG	Prescription change: intensification	NA	NA
21	N	Y	N	ROSUVASTATIN 40MG			ROSUVASTATI N 40MG			No prescription change	Pre not on goal	Post not on goal, decrease in Post
22	N	N	N	ATORVASTATIN 40 MG			ATORVASTATI N 80MG	EZETIMIBE 10MG		Prescription change: intensification	NA	NA
23	N	N	N	ATORVASTATIN 40MG			ATORVASTATI N 40MG			No prescription change	Pre not on goal	Post not on goal, decrease in Post
24	Y	Y	N	SIMVASTATIN 40MG			EZETIMIBE 10MG	PRAVASTATI N 20MG	EVOLOCUMAB 140MG	Prescription change: intensification	NA	NA
25	N	N	N	ROSUVASTATIN 40MG			ROSUVASTATI N 40MG			No prescription change	NA	NA
26	N	N	N	ATORVASTATIN 80MG			ATORVASTATI N 80MG			No prescription change	NA	NA
27	N	N	N	ROSUVASTATIN 40MG			ROSUVASTATI N 40MG			No prescription change	Pre on goal	Post on goal
28	Y	Y	N	ATORVASTATIN 40MG						No prescription	Pre not on goal	Post not on goal, increase in Post
29	N	Y	N	ATORVASTATIN 20MG			ATORVASTATI N 80MG	EZETIMIBE 10MG		Prescription change: intensification	Pre not on goal	Post on goal
30	N	N	N	ROSUVASTATIN 10MG			ROSUVASTATI N 20MG			Prescription change: intensification	NA	NA

31	N	N	Y	ROSUVASTATIN 20MG			ATORVASTATI N 40MG			Prescription change: No change in intensity	NA	NA
32	N	N	N	ROSUVASTATIN 40MG			ROSUVASTATI N 40MG			No prescription change	NA	NA
33	N	N	N	ROSUVASTATIN 5MG			ROSUVASTATI N 5MG			No prescription change	Pre not on goal	Post on goal
34	Y	N	N	ATORVASTATIN 80MG						No prescription	Pre not on goal	Post not on goal, decrease in Post
35	N	Y	N	ROSUVASTATIN 10MG	COLESTIPOL HCL 1G	EZETIMIBE 10MG	ROSUVASTATI N 10MG	COLESTIPOL HCL 1G	EZETIMIBE 10MG	Prescription change: decrease in intensity	Pre not on goal	Post not on goal, increase in Post
36	N	Y	N	ATORVASTATIN 40MG			ATORVASTATI N 40MG			No prescription change	Pre not on goal	Post not on goal, decrease in Post
37	N	Y	Y	ATORVASTATIN 80MG			ATORVASTATI N 80MG			No prescription change	Pre not on goal	Post not on goal, no change in Post
38	N	N	N	ATORVASTATIN 40MG			ATORVASTATI N 40MG	COLESTIPOL 1G		Prescription change: intensification	NA	NA
39	N	N	Y	ROSUVASTATIN 40MG			ROSUVASTATI N 40MG	EZETIMIBE 10MG		Prescription change: intensification	Pre not on goal	Post not on goal, no change in Post
40	N	N	N	ATORVASTATIN 40 MG			ROSUVASTATI N 40MG			Prescription change: No change in intensity	NA	NA
41	Y	Y	Y	ATORVASTATIN 80 MG			ATORVASTATI N 80MG	EVOLOCUMA B 140MG		Prescription change: intensification	Pre not on goal	Post on goal
42	Y	N	N	ROSUVASTATIN 40MG			ROSUVASTATI N 40MG			No prescription change	NA	NA
43	N	N	N	ATORVASTATIN 40MG			ATORVASTATI N 80MG			Prescription change: No change in intensity	Pre not on goal	Post on goal
44	N	N	N	ROSUVASTATIN 40MG			ROSUVASTATI N 40MG			No prescription change	Pre not on goal	Post not on goal, increase in Post
45	N	N	N	EZETIMIBE-SIMVASTATIN 10-40MG			EZETIMIBE-SIMVASTATIN 10-40MG			No prescription change	Pre on goal	Post on goal
46	N	N	N	ATORVASTATIN 10MG			ATORVASTATI N 10MG			No prescription change	Pre not on goal	Post not on goal, decrease in Post

47	N	Y	Y	ROSUVASTATIN 40MG						No prescription	Pre not on goal	Post not on goal, decrease in Post
48	N	N	N	ROSUVASTATIN 40MG			ROSUVASTATI N 40MG	EZETIMIBE 10MG		Prescription change: intensification	NA	NA
49	N	N	N	ATORVASTATIN 40MG			ATORVASTATI N 40MG			No prescription change	NA	NA
50	N	N	N	ATORVASTATIN 40MG			ATORVASTATI N 40MG			No prescription change	NA	NA
51	N	Y	N	ROSUVASTATIN 40MG			ROSUVASTATI N 40MG			No prescription change	Pre not on goal	Post on goal
52	Y	N	N	ATORVASTATIN 40MG			ATORVASTATI N 40MG			No prescription change	Pre on goal	Post on goal
53	N	Y	Y	ROSUVASTATIN 10MG	EZETIMIBE 10MG		EZETIMIBE 10MG			Prescription change: decrease in intensity	Pre not on goal	Post not on goal, no change in Post
54	Y	Y	N	ATORVASTATIN 20MG			ATORVASTATI N 80MG			Prescription change: intensification	Pre not on goal	Post not on goal, decrease in Post
55	N	N	N	ROSUVASTATIN 20MG			ROSUVASTATI N 20MG			No prescription change	Pre on goal	Post on goal
56	Y	Y	N	ATORVASTATIN 40MG						No prescription	Pre not on goal	Post not on goal, increase in Post
57	Y	Y	Y	PRAVASTATIN 80MG	COLESEVELAM 625MG		COLESEVELAM 625MG	ATORVASTA TIN 80MG		Prescription change: intensification	Pre not on goal	Post not on goal, no change in Post
58	Y	Y	N	SIMVASTATIN 10MG			EZETIMIBE 10MG			Prescription change: decrease in intensity	NA	NA
59	N	N	N	ROSUVASTATIN 20MG			ROSUVASTATI N 20MG			No prescription change	Pre not on goal	Post on goal
60	N	N	N	ROSUVASTATIN 20MG			ROSUVASTATI N 40MG			Prescription change: No change in intensity	Pre not on goal	Post not on goal, no change in Post
61	N	Y	N	ATORVASTATIN 10MG			ATORVASTATI N 10MG			No prescription change	NA	NA
62	N	Y	N	ATORVASTATIN 80MG			ATORVASTATI N 80MG			No prescription change	NA	NA
63	N	N	N	ATORVASTATIN 80MG			ATORVASTATI N 80MG			No prescription change	NA	NA
64	N	N	N	ATORVASTATIN 80MG	EZETIMIBE 10MG		ATORVASTATI N 80MG	EZETIMIBE 10MG		No prescription change	Pre not on goal	Post not on goal, decrease in Post

65	N	N	N				ATORVASTATI N 80mg			Prescription change: intensification	Pre not on goal	Post on goal
66	Y	N	N				ATORVASTATI N 40MG			Prescription change: intensification	Pre not on goal	Post not on goal, decrease in Post
67	N	Y	N				ROSUVASTATI N 40MG	EZETIMIBE 10MG		Prescription change: intensification	NA	NA
68	Y	N	N							No prescription	NA	NA
69	N	N	N	ATORVASTATIN 80MG						No prescription	NA	NA
70	N	N	N	ATORVASTATIN 40MG						No prescription	Pre not on goal	Post not on goal, decrease in Post
71	N	N	N	ROSUVASTATIN 40MG						No prescription	Pre not on goal	Post not on goal, decrease in Post
72	N	N	N	SIMVASTATIN 40MG						No prescription	NA	NA
73	N	N	N	ATORVASTATIN 80MG						No prescription	NA	NA
74	N	N	N							No prescription	NA	NA
75	Y	N	N	ATORVASTATIN 80MG						No prescription	NA	NA
76	N	N	N	ROSUVASTATIN 40MG			ROSUVASTATI N 40MG			No prescription change	NA	NA
77	N	N	N							No prescription	NA	NA
78	N	N	N							No prescription	NA	NA
79	N	N	N							No prescription	NA	NA
80	N	N	Y							No prescription	Pre on goal	Post not on goal
81	N	Y	N							No prescription	NA	NA
82	Y	N	N							No prescription	Pre not on goal	Post not on goal, decrease in Post
83	Y	N	N							No prescription	NA	NA
84	N	N	N							No prescription	NA	NA
85	N	N	N							No prescription	NA	NA
86	N	N	Y	ROSUVASTATIN 40MG			ROSUVASTATI N 40MG			No prescription change	NA	NA

87	N	N	N	ROSUVASTATIN 20MG	EZETIMIBE 10MG		ROSUVASTATI N 20MG	EZETIMIBE 10MG	EVOLOCUMAB 140MG/ML	Prescription change: intensification	NA	NA
88	Y	Y	N							No prescription	Pre not on goal	Post not on goal, decrease in Post
89	Y	Y	Y	MICRONIZED COLESTIPOL 1G	ROSUVASTATI N 40MG	EZETIMIBE 10MG	ROSUVASTATI N 40MG	EZETIMIBE 10MG		Prescription change: decrease in intensity	Pre not on goal	Post not on goal, decrease in Post
90	Y	N	N	EZETIMIBE 10MG	ATORVASTATI N 80MG		EZETIMIBE 10MG	ATORVASTA TIN 80MG		No prescription change	Pre on goal	Post on goal
91	N	N	Y	ROSUVASTATIN 20MG			ROSUVASTATI N 40MG	EZETIMIBE 10MG	EVOLOCUMAB 140MG/ML	Prescription change: intensification	NA	NA
92	Y	N	N	ATORVASTATIN 80MG			ATORVASTATI N 80MG			No prescription change	Pre not on goal	Post not on goal, decrease in Post
93	N	N	N	ROSUVASTATIN 10MG	FENOFIBRATE 145MG		ROSUVASTATI N 10MG			Prescription change: decrease in intensity	Pre not on goal	Post not on goal, no change in Post
94	N	N	N	EZETIMIBE 10MG	ATORVASTATI N 20MG		ATORVASTATI N 80MG	PRALUENT 75ML/MG		Prescription change: intensification	NA	NA
95	N	N	N	SIMVASTATIN 10MG						No prescription	NA	NA
96	Y	N	N	EZETIMIBE 10MG	ROSUVASTATI N 40MG		EZETIMIBE 10MG	ROSUVASTA TIN 40MG		No prescription change	NA	NA

Only includes values for participants who had both pre- and post-disclosure LDL-C values available for review and the post-disclosure LDL-C reflects the most recent prescription, i.e. LDL-C level had to be tested at least 4 weeks after changing the prescription. Pre-disclosure LDL-C was defined as the most recent LDL-C drawn prior to the disclosure of the FH-associated variant. Post-disclosure LDL-C was defined as the last LDL-C drawn before the end of the study period. LDL-C on goal was targeted at < 100 mg/dL for those treatment for primary prevention and < 70 mg/dL for secondary prevention. Decrease and increase change in post-disclosure is only counted as valid change when % of LDL-C level change is >=8%.Y=Yes; N=No; NA=Not Available; MI=myocardial infarction; LDL-C= low-density lipoprotein cholesterol.
